# Critical windows of exposure to air pollution and gestational diabetes: assessing effect modification by maternal pre-existing conditions and environmental factors

**DOI:** 10.1186/s12940-023-00974-z

**Published:** 2023-03-15

**Authors:** Marcel Miron-Celis, Robert Talarico, Paul J. Villeneuve, Eric Crighton, David M. Stieb, Cristina Stanescu, Éric Lavigne

**Affiliations:** 1 Air Sectors Assessment and Exposure Science Division, Health Canada, Ottawa, ON Canada; 2 ICES uOttawa (Formerly Known As Institute for Clinical Evaluative Sciences), Ottawa, ON Canada; 3 Ottawa Hospital Research Institute, The Ottawa Hospital, Ottawa, ON Canada; 4 Department of Neuroscience, Carleton University, Ottawa, Canada; 5 Department of Geography, Environment and Geomatics, University of Ottawa, Ottawa, ON Canada; 6 Population Studies Division, Health Canada, 269 Laurier Avenue West, Ottawa, ON K1A 0K9 Canada; 7 School of Epidemiology and Public Health, University of Ottawa, Ottawa, ON Canada

**Keywords:** Gestational Diabetes, Air pollution, Pre-existing conditions, Green Space, Pregnancy

## Abstract

**Background:**

Ambient air pollution has been associated with gestational diabetes (GD), but critical windows of exposure and whether maternal pre-existing conditions and other environmental factors modify the associations remains inconclusive.

**Methods:**

We conducted a retrospective cohort study of all singleton live birth that occurred between April 1^st^ 2006 and March 31^st^ 2018 in Ontario, Canada. Ambient air pollution data (i.e., fine particulate matter with a diameter ≤ 2.5 μm (PM_2.5_), nitrogen dioxide (NO_2_) and ozone (O_3_)) were assigned to the study population in spatial resolution of approximately 1 km × 1 km. The Normalized Difference Vegetation Index (NDVI) and the Green View Index (GVI) were also used to characterize residential exposure to green space as well as the Active Living Environments (ALE) index to represent the active living friendliness. Multivariable Cox proportional hazards regression models were used to evaluate the associations.

**Results:**

Among 1,310,807 pregnant individuals, 68,860 incident cases of GD were identified. We found the strongest associations between PM_2.5_ and GD in gestational weeks 7 to 18 (HR = 1.07 per IQR (2.7 µg/m^3^); 95% CI: 1.02 – 1.11)). For O_3_, we found two sensitive windows of exposure, with increased risk in the preconception period (HR = 1.03 per IQR increase (7.0 ppb) (95% CI: 1.01 – 1.06)) as well as gestational weeks 9 to 28 (HR 1.08 per IQR (95% CI: 1.04 –1.12)). We found that women with asthma were more at risk of GD when exposed to increasing levels of O_3_ (*p*- value for effect modificatio*n* = 0.04). Exposure to air pollutants explained 20.1%, 1.4% and 4.6% of the associations between GVI, NDVI and ALE, respectively.

**Conclusion:**

An increase of PM_2.5_ exposure in early pregnancy and of O_3_ exposure during late first trimester and over the second trimester of pregnancy were associated with gestational diabetes whereas exposure to green space may confer a protective effect.

**Supplementary Information:**

The online version contains supplementary material available at 10.1186/s12940-023-00974-z.

## Introduction

Gestational diabetes is one of the most common pregnancy complications, affecting approximately 3–5% of pregnancies [1]. It is also a substantial contributor to both maternal and neonatal morbidity and mortality in most countries [2–5]. The specific disease pathways involved in the pathophysiology of gestational diabetes are yet to be clearly elucidated, but it is commonly argued that these conditions are multifactorial — often involving genetic, social and environmental factors [6]. On the environmental front, ambient air pollution has been associated with gestational diabetes [7]. While the underlying mechanism(s) of this association are not well understood, evidence suggests that exposure to air pollution during pregnancy can lead to oxidative stress and inflammatory processes that increase the likelihood of gestational diabetes [7]. Recent evidence has also highlighted the importance of critical air pollution exposure windows, with maternal exposure in the 21^st^ to 24^th^ gestational weeks posing particular risks [8]. Few studies, however, have assessed the preconception period as a vulnerable window for gestational diabetes [7]. In addition, associations between ambient air pollution and gestational diabetes among those with pre-existing medical conditions are not well understood [9, 10]. This is important because the presence of inflammation is also a common characteristic among individuals with pre-existing medical conditions such as asthma and hypertension. The role of other environmental factors including features of the natural and built environments (and their interrelationship with ambient air pollution) in the etiology of gestational diabetes requires further examination.

While there is a growing body of evidence pointing to the relationships between natural and built environments in determining diverse health outcomes including birth weight and preterm delivery [11, 12], few studies have examined their relationship to gestational diabetes or in particular how these environments may interact with exposure to ambient air pollution during pregnancy [13, 14]. For instance, there is a considerable research gap relating to how natural and built environment characteristics (e.g., access to green environment and neighborhood friendliness to active living – “walkability”) may modify associations between ambient air pollution exposure and gestational diabetes. Greenness exposure during pregnancy has been explored extensively for its association with fetal growth, birthweight, gestational age, preterm birth and head circumference, and it has generally been found to have protective effects against adverse outcomes, though results are not entirely consistent [15]. However, only a small amount of researches has investigated the interrelationship between green space, air pollution and gestational diabetes [13, 14]. For example, one can hypothesize that green space may confer a protective effect against gestational diabetes by reducing exposure to air pollution, which may be associated to a possible mediation effect that has been observed on other maternal and pregnancy outcomes [16]. Greenness has also been associated to a reduction of stress, an increase of opportunity for physical activity and their combined effect could reduce the risk of gestational diabetes. Associations between exposures to air pollution and gestational diabetes could also be impacted by neighborhood walkability, in which highly walkable neighbourhoods are associated with higher levels of physical activity, but likely more exposure to air pollution [17]. These factors may also reduce allostatic load [18–20].

Therefore, this study sought to investigate whether ambient air pollution increases the risk of gestational diabetes, accounting for pre-existing maternal health conditions, and assessing variations in risk across different exposure periods. In addition, the study investigates the extent to which associations between exposures to air pollution and gestational diabetes are modified by neighbourhood green space and active living friendliness. Given its impact on maternal and neonatal health, there is an important need to understand the etiological pathways of gestational diabetes to mitigate adverse impacts in both the mother and child.

## Material and methods

### Study design and population

We conducted a retrospective cohort study using data on singleton live births that occurred between April 1^st^ 2006 and March 31^st^ 2018 in the Province of Ontario, Canada. Mother-infant pairs were obtained from the Better Outcomes Registry & Network (BORN) Ontario, a province wide birth registry that captures perinatal health information (https://www.bornontario.ca/en/about-born/). Data pertaining to each hospital birth in Ontario are collected from patient charts by hospital staff from clinical forms, and patient interviews, and then entered into the BORN information system. The registry contains information on maternal demographics, health behaviours (e.g. smoking, alcohol use), reproductive history, and clinical information related to pregnancy, labour, birth, and foetal and neonatal outcomes. Formal training of data collectors and ongoing data validation programs ensure the database is maintained with high quality data. We used the Postal Code Conversion File Plus (PCCF +) to obtain the geographic coordinates of maternal place(s) of residence based on residential postal code(s) reported in health administrative data. Pregnancies with postal codes of residence outside Ontario were excluded from the analysis. Subjects with pre-gestational diabetes (type 1 or type 2), without a valid health card number, missing date of birth, missing information on the sex of the new-born, postal code value, or mothers who did not have continuous residence in Ontario, Canada for their respective gestational period were excluded. A flow chart describing the exclusion process in presented in Supplementary Fig. 1. As well, we had no information on birth outcomes for some women who were pregnant at the same time as participants of our study, but gave birth before the study started or after the study ended. In order to account for the non-inclusion of these women, which has been described previously as the “fixed cohort bias” [21], we included only births with estimated conception dates ranging from 20 weeks (i.e. shortest pregnancy) before the study started to 44 weeks (i.e. longest pregnancy) before it ended.

### Outcome ascertainment

Incident cases of gestational diabetes were obtained from the BORN registry [22] between April 1^st^, 2006 and March 31^st^, 2018. From 2006 through 2013, gestational diabetes was diagnosed in Ontario using the 2003 and 2008 Canadian Diabetes Association’s guidelines, while the updated 2013 guidelines were used from 2013 to 2018 [23]. The 2003 and 2008 guidelines recommend universal screening for gestational diabetes using a 50 g glucose challenge test (GCT) at 24–28 weeks gestation, and when positive (i.e., > 7.8 mmol/L), a subsequent 75 g oral glucose tolerance test (OGTT) was needed to confirm the presence of gestational diabetes (positive thresholds: fasting, ≥ 5.3 mmol/L; 1 h, ≥ 10.6 mmol/L; 2 h, ≥ 8.9 mmol/L). Gestational diabetes was diagnosed when the results showed ≥ 2 positive OGTT results or a GCT result ≥ 10.3 mmol/L. The updated 2013 guidelines proposed two diagnostic methods to ascertain gestational diabetes. The first method was nearly identical to the 2003/2008 guidelines with only a slight increase in some of the positive threshold values (i.e., 50 g GCT ≥ 11.1 mmol/L and 2 h OGTT ≥ 9.0 mmol/L). The second method was a one-step approach involving only the 75 g OGTT with the updated positive threshold values.

### Exposure ascertainment

Ambient air pollution during pregnancy was the primary exposure of interest. Residential exposures to fine particulate matter with a diameter ≤ 2.5 μm (PM_2.5_), nitrogen dioxide (NO_2_) and ozone (O_3_) were assigned to the study population at the geographic centre of each 6-digit postal code area. This assignment was facilitated by the Postal Code Conversion File Plus which was used to convert residential postal codes into geographic coordinates [24]. The mother’s residences during pregnancy were used for determining exposure assignment during pregnancy. We used air pollution surfaces available at spatial resolutions of approximately 1-km^2^. The PM_2.5_ surface was derived using satellite-based estimates that were combined with ground-level monitor information and chemical transport models, as described by van Donkelaar et al. [25]. NO_2_ was assessed based on a national land-use regression (LUR) model, using data from the Canadian National Air Pollution Surveillance (NAPS) monitoring network, combined with information on satellite-derived NO_2_ estimates, road lengths within 10 km (km), area of industrial land use within 2 km and the mean summer rainfall [26]. O_3_ was assessed based on a surface that represents an average of daily 8 h maximum concentrations in the warm seasons (May 1st to October 31st) using an optimal interpolation technique described previously [27]. PM_2.5_ levels are described in micrograms per cubic meter (µg/m^3^) while O_3_ and NO_2_ levels are described in parts per billion (ppb). Air pollution estimates were available on a weekly level from April 1^st^ 2006 until March 31^st^ 2018, based on temporal scaling previously described [28]. Therefore, exposures were assigned for each week of pregnancy and for the preconception period (i.e. 12 weeks before estimated conception).

Other environmental exposure variables were also obtained from CANUE, namely, residential exposure to green space, noise and neighbourhood active living friendliness. Detailed description of the ascertainment to residential exposure to green space using the Normalized Difference Vegetation Index (NDVI) and the Green View Index (GVI) are provided in supplementary material. Estimation procedures for noise are described in detail elsewhere [29]. Noise is reported in A-weighted decibels (dBA). The Active Living Environments (ALE) index represents the active living friendliness of Canadian communities on a scale from 1 (very low) to 5 (very high). A negative value indicates below average active living friendliness, a positive value indicates above average active living friendliness, and a value of zero indicates average active living friendliness. We also extracted data on daily average ambient temperature throughout the study period from the Daymet dataset at a 1 km × 1 km grid spatial resolution across Canada [30]. The data were then converted into weekly averages to match the air pollution data.

The corresponding values of all exposure variables were assigned to each cohort member using the centroid geographical coordinates of the home address postal code. The exposure data were linked to the study cohort and analyzed by the Institute of Clinical Evaluative Science (ICES). ICES is an independent, non-profit research institute funded by an annual grant from the Ontario Ministry of Health and Long-Term Care (MOHLTC). As a prescribed entity under Ontario’s privacy legislation, ICES is authorized to collect and use health care data for the purposes of health system analysis, evaluation and decision support. Secure access to these data is governed by policies and procedures that are approved by the Information and Privacy Commissioner of Ontario.

### Covariates

Covariates were available from BORN and included maternal age at delivery, maternal cigarette smoking anytime during pregnancy, parity, pre-pregnancy body mass index, month of birth and year of birth. We also captured gestational age, which was determined from the mother’s last menstrual period and ultrasound dating. Several additional covariates were also derived based on individual’s postal code(s) of residence during pregnancy: (1) a dichotomous variable classifying Ontario into the Greater Toronto Area, a densely-populated urban mega-region, and all other areas; (2) a categorical variable classifying the size of the community where individuals lived; (3) area-level deprivation based on the Ontario Marginalization Index, which quantifies the degree of marginalization between areas and inequalities in health and social well-being in Ontario and includes deprivation quintiles, instability quintiles, ethnic quintiles and dependency quintiles [31].

A directed acyclic graph (DAG) was conceptualized using previous knowledge on potential confounders. Using this approach the following covariates were included in all statistical models: maternal age, parity, maternal smoking status, pregnancy body mass index, weekly ambient mean temperatures, month of birth, year of birth, residence in the Greater Toronto Area, community size, and the Ontario Marginalization Index. The conceptual DAG showing the pathways through which these variables may influence the exposures and the outcomes of interest is shown in Supplementary Fig. 2.

Pre-pregnancy health conditions (i.e. conditions present before pregnancy) among pregnant individuals considered as potential effect modifiers in the investigated associations included asthma, and hypertension, Information on pre-existing health conditions was captured from BORN.

### Statistical analysis

Multivariable Cox proportional hazards regression models were used to evaluate the associations between each of the three air pollutants as continuous variables and gestational diabetes. We used gestational weeks of pregnancy as the underlying timescale in the Cox models. Follow-up was conducted from the 20^th^ week of gestation until gestational diabetes diagnosis, delivery, still birth, maternal death or loss of eligibility for provincial health insurance. Results are expressed as the hazard ratio (HR) and 95% confidence interval (CI) corresponding to an increase across the interquartile range (IQR) of the pollutant of interest.

An extension of the distributed lag non-linear model (DLNM) was used to simultaneously investigate exposure by preconception weeks as well as by each of the first 37 weeks during pregnancy [32]. This method allows for identification of critical windows of exposure for complex exposure–response relationships [33]. To select the appropriate model, different lag structures (natural and B splines) and number of knots (2–5 knots) were used to define the crossbasis of pregnancy exposures. The crossbasis that minimized the Akaike Information Criterion (AIC) was selected as the final model. Estimates of association were obtained by calculating the cumulative hazard over the preconception period, pregnancy period and critical windows identified.

Next, effect modification by residential green space, ALE and maternal pre-existing health conditions were tested by including an interaction term between each air pollutant of interest and these variables. Wald’s method was used to assess the presence of interaction on the multiplicative scale. Effect modification was considered statistically significant if the interaction term *p*-value was less than 0.05. We also conducted a sensitivity analysis by limiting the follow-up to the end of the 28^th^ week of pregnancy since most women are being tested for gestational diabetes in Canada by the end of that week. We also additionally adjusted models for noise pollution. A mediation analysis was also conducted to assess whether the effects of exposure to green spaces and ALE might be mediated by air pollution. We reported natural direct, indirect and total effect of the impact of NDVI, GVI and ALE on gestational diabetes. Statistical analyses were conducted using R version 3.0.1,(R Core Team, 2019) using the *survival* (version 2.42–3), *dlnm* (version 2.1.3) and *medflex* packages.

## Results

A total of 1,310,807 pregnant individuals were included in the study cohort. From April 1^st^, 2006 to March 31^st^, 2018, a total of 68,860 incident cases of gestational diabetes were identified in the province of Ontario. The complete baseline characteristics of the study population are shown in detail in Table 1. Some differences on key demographic characteristics can be noted among those with vs. without incident gestational diabetes. Namely, individuals with gestational diabetes tended to be slightly older, have a higher pre-pregnancy BMI and a higher parity. Additionally, those with gestational diabetes had a lower prevalence of smoking during pregnancy and were more likely to reside in the Greater Toronto Area and live within a larger community. There were also notable differences in socio-economic status between those with and without incident gestational diabetes, as shown by the deprivation and ethnic concentration quintiles. The mean concentrations of PM_2.5_ and NO_2_ were slightly higher among women with gestational diabetes, while levels were similar across the two groups for O_3_.

**Table 1:** Descriptive characteristics of the study population (*N* = 1,310,807) at birth stratified by disease status

Characteristics	Presence of gestational diabetes *N* = 68,860	Absence of gestational diabetes *N* = 1,241,947
***Demographic & behavioural factors***		
Maternal age, years (Mean ± SD)	32.72 ± 5.06	30.14 ± 5.43
Gestational age, weeks (Mean ± SD)	38.22 ± 1.60	38.91 ± 1.78
Prepregnancy BMI (Mean ± SD)	28.50 ± 8.10	25.71 ± 7.09
Parity		
0	16,359 (38.7%)	555,150 (44.7%)
1	15,564 (36.9%)	452,069 (36.4%)
≥ 2	10,304 (24.4%)	234,728 (18.9%)
Smoking Status During Pregnancy		
Missing	1,079 (1.6%)	12,996 (1.0%)
No	62,135 (90.2%)	1,093,557 (88.1%)
Yes	5,646 (8.2%)	135,394 (10.9%)
Maternal pre-existing conditions		
Asthma	2,249 (3.3%)	44,090 (3.6%)
Hypertension	1,269 (1.8%)	7,889 (0.6%)
***Neighbourhood socio-economic factors***		
Instability		
Missing	1,052 (1.5%)	14,516 (1.2%)
1^st^ quintile	17,540 (25.5%)	264,609 (21.3%)
2^nd^ quintile	11,721 (17.0%)	231,123 (18.6%)
3^rd^ quintile	10,751 (15.6%)	215,826 (17.4%)
4^th^ quintile	12,072 (17.5%)	232,501 (18.7%)
5^th^ quintile	15,724 (22.8%)	283,372 (22.8%)
Dependency		
Missing	1,052 (1.5%)	14,516 (1.2%)
1^st^ quintile	25,234 (36.6%)	403,903 (32.5%)
2^nd^ quintile	15,262 (22.2%)	265,568 (21.4%)
3^rd^ quintile	11,168 (16.2%)	214,494 (17.3%)
4^th^ quintile	8,889 (12.9%)	183,931 (14.8%)
5^th^ quintile	7,255 (10.5%)	159,535 (12.8%)
Deprivation		
Missing	1,052 (1.5%)	14,516 (1.2%)
1^st^ quintile	10,351 (15.0%)	236,802 (19.1%)
2^nd^ quintile	10,952 (15.9%)	228,773 (18.4%)
3^rd^ quintile	12,521 (18.2%)	231,539 (18.6%)
4^th^ quintile	14,502 (21.1%)	237,554 (19.1%)
5^th^ quintile	19,482 (28.3%)	292,763 (23.6%)
Ethnic Concentration		
Missing	1,052 (1.5%)	14,516 (1.2%)
1^st^ quintile	5,725 (8.3%)	162,917 (13.1%)
2^nd^ quintile	6,656 (9.7%)	180,767 (14.6%)
3^rd^ quintile	8,532 (12.4%)	205,411 (16.5%)
4^th^ quintile	13,180 (19.1%)	260,678 (21.0%)
5^th^ quintile	33,715 (49.0%)	417,658 (33.6%)
***Geographical & Environmental factors***		
Community Size		
≥ 1 500 000	40,033 (58.1%)	564,514 (45.5%)
500 000–1 499 999	8,001 (11.6%)	167,535 (13.5%)
100 000–499 999	13,100 (19.0%)	292,069 (23.5%)
10 000–99 999	3,075 (4.5%)	91,510 (7.4%)
< 10 000	4,621 (6.7%)	125,945 (10.1%)
Missing	30 (0.0%)	374 (0.0%)
Greater Toronto Area residence		
Yes	28,008 (66.3%)	690,523 (55.6%)
No	14,219 (33.7%)	551,424 (44.4%)
Active Living Environment (Mean ± SD)	1.25 ± 3.52	0.69 ± 2.96
PM_2.5_, µg/m^3^ (Mean ± SD)	8.09 ± 1.58	7.97 ± 1.72
NO_2_, ppb (Mean ± SD)	13.23 ± 5.59	11.36 ± 5.40
O_3_, ppb (Mean ± SD)	48.32 ± 4.93	48.45 ± 4.92
NDVI (Mean ± SD)	0.69 ± 0.08	0.69 ± 0.08
Segmented GVI (Mean ± SD)	13.45 ± 8.05	13.95 ± 8.44
Ambient temperature	8.59 ± 3.06	8.30 ± 2.97
Noise, dB(A) (Mean ± SD)	59.38 ± 5.11	59.31 ± 5.02

The IQRs for PM_2.5_, NO_2_ and O_3_ over the entire preconception and gestational periods were 2.7 μg/m^3^, 10.02 ppb and 7.0 ppb, respectively (Supplementary Table 1). During the entire pregnancy, exposure to PM_2.5_ was weakly correlated with exposures to NO_2_ (*r* = 0.44) and O_3_ (*r* = -0.25) and the correlation between exposure to NO_2_ and O_3_ (*r* = -0.27) was weakly negative (all three significant at p < 0.001) (Supplementary Table S2). As well, there was a weak correlation between the two different green space metrics (i.e. GVI and NDVI) (*r* = 0.14).

**Fig. 1:**
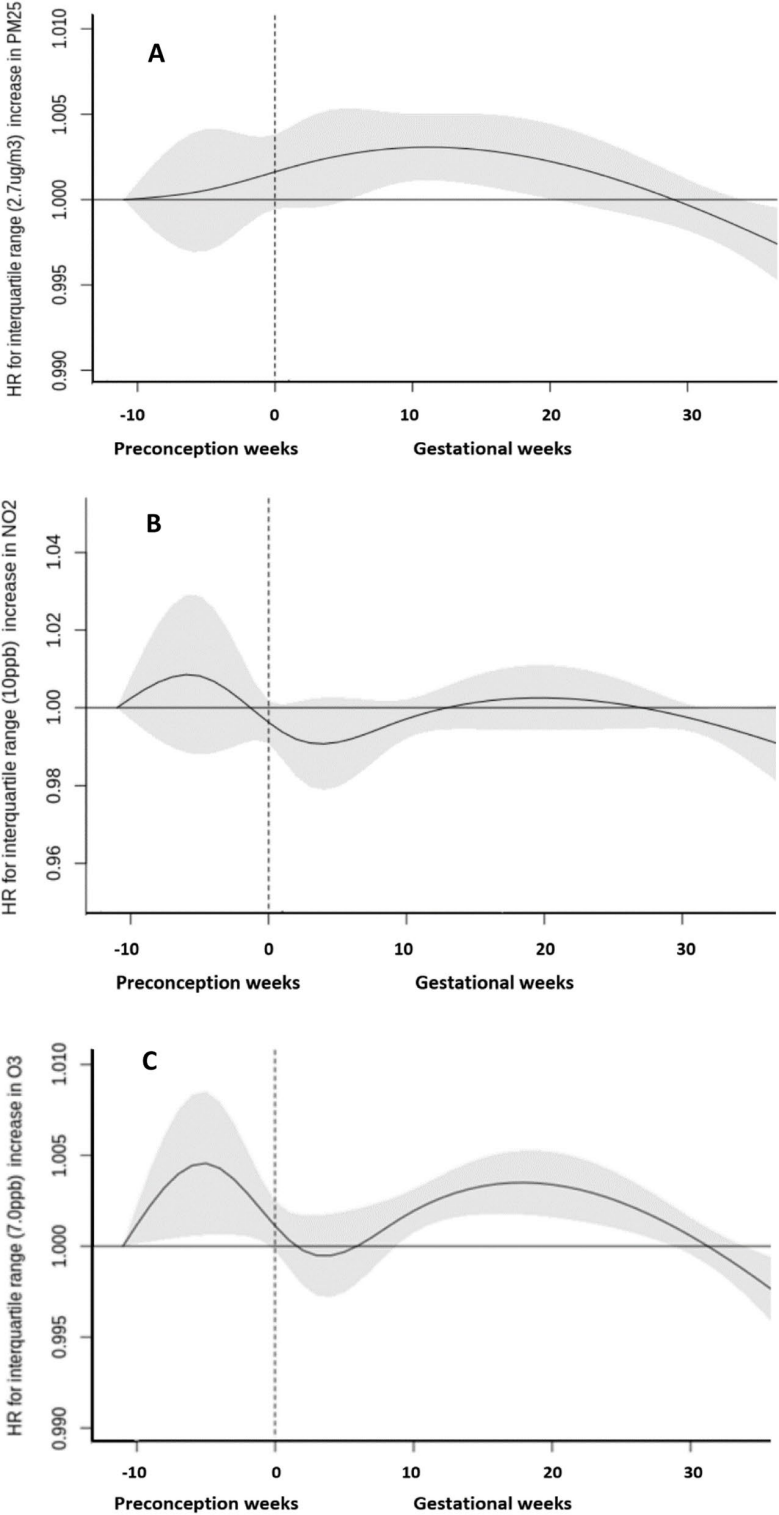
Weekly associated hazard ratios (HRs) associated with weekly PM_2.5_, NO_2_, and O_3_ exposures over the preconception period and the gestational period with risk of gestational diabetes in the overall cohort (*n* = 1,310,807). Gray shade indicates 95% confidence intervals; dashed vertical line demarcate preconception and post conception weeks. All the models were adjusted for maternal age, parity, maternal smoking status, prepregnancy body mass index, weekly ambient temperatures, month of birth, year of birth, residing in the Greater Toronto Area, community size, deprivation quintiles, instability quintiles, dependency quintiles and ethnic quintiles

Associations between weekly exposures to air pollutants and gestational diabetes for identifying potential sensitive windows are presented in Fig. 1. Associations between PM_2.5_ and gestational diabetes appeared to be strongest and most highly statistically significant from weeks 7 to 18 during the gestational period. The cumulative HR for those weeks of gestation was 1.07 per IQR (2.7 µg/m^3^) increase in PM_2.5_ (95% CI: 1.02 – 1.11) (Table 2). We did not identify a sensitive window for weekly exposures to NO_2_. The cumulative HRs over the preconception and gestational periods for NO_2_ exposure were 1.05 per IQR (10.0 ppb) increase (95% CI: 0.91, 1.21) and 0.99 (95% CI: 0.85, 1.16), respectively (Table 2). For O_3_, we found two sensitive windows of exposure, with statistically significant increased risk in the preconception period as well as gestational weeks 9 to 28. The cumulative HR for the sensitive window during the preconception period was 1.03 per IQR increase (7.0 ppb) (95% CI: 1.01 – 1.06). The cumulative HR for the sensitive window of 9–28 weeks of gestation was 1.08 per IQR increase (95% CI: 1.04, 1.12) (Table 2). For all three pollutants, the cumulative HR over the whole gestational period was not statistically significant. We did not find meaningful differences in the HRs when adjusting only for individual-level covariates (Supplementary Table 2) as opposed to adding neighborhood level covariates in the models as presented in Table 2.

**Table 2:** Adjusted^a^ cumulative hazard ratios (HRs) and 95% confidence intervals (CIs) of gestational diabetes per interquartile range (IQR) increase in PM_2.5_, NO_2_, and O_3_ for the preconception period, entire pregnancy and DLM-identified sensitive windows

Pollutant	HR (95% CI)
**PM**_**2.5**_** (per IQR = 2.7 µg/m**^**3**^** increase)**	
Preconception period	1.01 (0.98 – 1.03)
Pregnancy period	1.05 (1.00 – 1.09)
Sensitive windows	1.07 (1.02 – 1.11)
**NO**_**2**_** (per IQR = 10.0 ppb increase)**	
Preconception period	1.07 (0.88 – 1.23)
Pregnancy period	1.00 (0.85 – 1.16)
Sensitive windows	-
**O**_**3**_** (per IQR = 7.0 ppb increase)**	
Preconception period	1.03 (1.01 – 1.06)
Pregnancy period	1.04 (1.00 – 1.08)
Sensitive windows	1.08 (1.04 – 1.12)

^a^Adjusted for maternal age, parity, maternal smoking status, prepregnancy body mass index, weekly ambient temperatures, month of birth, year of birth, residing in the Greater Toronto Area, community size, deprivation quintiles, instability quintiles, dependency quintiles and ethnic quintiles

We found evidence of effect modification by maternal asthma status for exposure to O_3_ over the sensitive window from the 9^th^ to 28^th^ weeks of gestation (*p* value for effect modificatio*n* = 0.041) (Table 3). For instance, a cumulative HR of 1.12 (95% CI: 1.07 – 1.15) per IQR increase (7.0 ppb) for gestational diabetes was observed among women with asthma. In comparison, women without asthma had a cumulative HR of 1.04 (95% CI: 1.01 – 1.07). We also found that the cumulative HR for gestational diabetes for the sensitive window of exposure to PM_2.5_ was higher among women with asthma, but the effect modification was not statistically significant (*p* value for effect modificatio*n* = 0.099). We did not find differences across other characteristics investigated, although cumulative HRs appeared higher in neighbourhoods with elevated levels of ALE index.

**Table 3:** Adjusted^a^ cumulative hazard ratios (HRs) and 95% confidence intervals (CIs) of gestational diabetes per interquartile range (IQR) increase in PM_2.5_ and O_3_ for the DLM-identified sensitive windows, stratified by potential effect modifiers

Potential effect modifiers	PM_2.5_ 7^th^ to 18^th^ weeks of gestation	O_3_ Preconception weeks	O_3_ 9^th^ to 28^th^ weeks of gestation
	**HR (95% CI)**	**HR (95% CI)**	**HR (95% CI)**
Asthma			
Presence	1.21 (1.05 – 1.35)	0.96 (0.88 – 1.04)	1.12 (1.07 – 1.15)
Absence	1.06 (1.01 – 1.10)	1.03 (1.01 – 1.06)	1.04 (1.01 – 1.07)
*P* value for effect modification	0.099	0.331	0.041
Chronic hypertension			
Presence	0.95 (0.80 – 1.14)	0.96 (0.86 – 1.08)	1.03 (0.88 – 1.21)
Absence	1.07 (1.02 – 1.11)	1.00 (0.99 – 1.02)	1.08 (1.04 – 1.12)
*P* value for effect modification	0.754	0.852	0.842
NDVI			
1^st^ tertile	1.10 (1.06 – 1.14)	1.01 (0.98 – 1.04)	1.05 (1.02 – 1.09)
2^nd^ tertile	1.07 (1.02 – 1.11)	1.03 (1.01 – 1.06)	1.08 (1.04 – 1.12)
3^rd^ tertile	1.04 (1.00 – 1.08)	1.00 (0.98 – 1.03)	1.04 (1.01 – 1.06)
*P* value for effect modification	0.423	0.732	0.891
GVI			
1^st^ tertile	1.12 (1.07 – 1.16)	1.00 (0.97 – 1.03)	1.02 (0.98 – 1.06)
2^nd^ tertile	1.06 (1.02 – 1.10)	1.01 (0.98 – 1.04)	1.06 (1.02 – 1.10)
3^rd^ tertile	1.07 (1.02 – 1.11)	1.03 (1.00 – 1.06)	1.09 (1.04 – 1.13)
*P* value for effect modification	0.521	0.789	0.652
Active Living Environment			
1^st^ tertile	1.06 (1.01 – 1.10)	1.00 (0.97 – 1.04)	1.03 (0.99 – 1.07)
2^nd^ tertile	1.02 (0.98 – 1.06)	1.01 (0.99 – 1.04)	1.06 (1.02 – 1.10)
3^rd^ tertile	1.12 (1.08 – 1.16)	1.03 (1.00 – 1.05)	1.08 (1.04 – 1.12)
*P* value for effect modification	0.489	0.921	0.356

^a^Adjusted for maternal age, parity, maternal smoking status, prepregnancy body mass index, weekly ambient temperatures, month of birth, year of birth, residing in the Greater Toronto Area, community size, deprivation quintiles, instability quintiles, dependency quintiles and ethnic quintiles

In sensitivity analyses, we investigated the mediating effects of PM_2.5_, NO_2_ and O_3_ in the associations between independent exposures to GVI, NDVI and ALE on gestational diabetes (Supplementary Table 4). We found that exposure to air pollutants explained 20.1%, 1.4% and 4.6% of the associations between GVI, NDVI and ALE, respectively. In addition, adjusting for noise exposure at the place of residence of pregnant individuals did not modify substantially the HRs (data not shown).

## Discussion

Findings from this large population-based birth cohort study showed that exposures to PM_2.5_ and O_3_ during early to mid-pregnancy increased the risk of gestational diabetes. Preconception exposure to O_3_ appeared to increase the risk of gestational diabetes. We also found evidence that the presence of pre-pregnancy maternal asthma increased susceptibility to the impact of exposure to O_3_ during pregnancy on the incidence of gestational diabetes. We did not find evidence that pre-pregnancy maternal hypertension or the investigated environmental factors modified susceptibility to air pollution for gestational diabetes.

Several epidemiological studies have examined the associations between ambient air pollution and the risk of gestational diabetes [7, 10, 34]. However, few studies have investigated critical windows of exposure on a weekly level during preconception and gestational periods. In a recent study applying similar methods to ours conducted in China, Chen et al. found that exposures to PM_2.5_ among 4174 pregnant women during the 21^st^ to 24^th^ gestational weeks was the most critical window of exposure for increasing the risk of gestational diabetes [35]. In a meta-analysis of 11 epidemiological studies, authors found that second trimester PM_2.5_ exposure was associated with increased gestational diabetes risk (OR = 1.04, 95% CI: 1.01 – 1.09, per 10 μg/m^3^ increase in PM_2.5_) [7]. In fact, a recent study conducted in California, which used monthly estimates of ambient air pollutants, found stronger associations of PM_2.5_ exposure during the second trimester with gestational diabetes, except for black carbon which was more strongly associated with gestational diabetes during early pregnancy [9]. In our study, we found that PM_2.5_ exposure during the 7^th^ to 18^th^ gestational weeks (i.e. overlapping late first trimester and early second trimester) appeared to be the most important critical window.

We also observed positive associations between O_3_ exposures during the preconception period as well as during gestational weeks 9 to 28, and incidence of gestational diabetes. Results from the meta-analysis by Zhang et al. that included 13 epidemiological studies showed that prepregnancy O_3_ exposure was inversely associated with gestational diabetes (OR = 0.98, 95% CI: 0.98–0.99) while no associations were observed for trimester 1 or 2 exposures [34]. Evidence for exposure to NO_2_ during different trimesters has been inconclusive according to recent meta-analyses [7, 34], except for first trimester exposure to nitrogen oxides (NO_x_) which appeared to be associated with gestational diabetes [7]. However, in a study among 395,927 pregnancies in southern California, authors found that NO_2_ was the pollutant most strongly associated with gestational diabetes [9]. In our study, we did not find any association with exposure to NO_2_. Further research is needed, in particular in understanding specific components of particulate matter and mixtures of pollutants driving those risks during finely resolved (i.e. weekly) critical windows of exposure.

In terms of potential biological mechanisms, previous evidence in animals has shown that PM_2.5_ can affect glucose homeostasis, metabolic inflammatory responses, the production of reactive oxygen species, insulin resistance and glucose tolerance [36]. Exposure to PM_2.5_ during the later part of the first trimester and part of the second trimester could induce increases in fasting plasma glucose which can increase the likelihood of gestational diabetes diagnosis [37]. The fact that we found some effects during the preconception period could be explained by the fact that previous studies have shown that air pollution exposure before conception in rodents has led to adipose tissue inflammation and the generation of reactive oxygen species which may result in insulin resistance [38].

We observed a higher risk of gestational diabetes among pregnant individuals with asthma exposed to O_3_ during late first trimester and throughout the second trimester. Prior literature has shown that inhalation of gaseous pollutants can induce pro-inflammatory processes during pregnancy [39]. Inflammation is also a characteristic feature of the pathophysiology of asthma [40]. It is therefore biologically plausible that inflammation from exposure to air pollution during pregnancy combined with inflammation due to maternal presence of asthma increases the risk of gestational diabetes. These findings require further investigation.

A mediation analysis was also done to explore the etiological pathways of the green space metrics (i.e. GVI and NDVI) and a measure of neighbourhood active living friendliness (i.e. ALE). The results showed that air pollution exposure explained 20.1%, 1.4% and 4.6% of the effects of GVI, NDVI and the ALE on the development of gestational diabetes, respectively. Evidence from a study conducted in Wuhan, China, showed that exposure to PM_2.5_ also mediated the association between residential green space exposure during pregnancy and development of gestational diabetes [14]. In our study, we found that of neighbourhood built environment measures, the effect of GVI on incidence of gestational diabetes was most strongly mediated by air pollution. The GVI metric could potentially capture exposure to trees to a better extent than NDVI, which may have a stronger impact on reducing air pollution levels. In fact, assessing green space exposure with street view images is a novel method and its advantages are being identified. Similarly to correlations found in this study, Larkin and colleagues found low correlations between GVI and NDVI [41]. This preliminary evidence requires further investigation.

One important strength of this study is its very large sample size, as it allows for greater sensitivity to detect the effects of specific exposures while allowing adjustment for numerous potential confounders. Secondly, the rich individual-level covariate data strengthen the internal validity of the study and renders the results less prone to residual confounding. Thirdly, our methodology allowed the identification of critical windows of exposure rather than averaging exposures by trimesters in order to account for potentially different periods of vulnerability during pregnancy. Some limitations should also be considered. Some of the data used for this study came from administrative sources, which may be less accurate than clinical data. Additionally, estimates for exposures of interest were not ascertained at the level of full address of residence, but rather at the six character postal code level, potentially introducing exposure measurement error. Finally, the medical diagnostic criteria of gestational diabetes considered in this study changed during the 12-year study period, which could influence the incidence rates of the outcomes and affect the results in unpredictable ways.

## Conclusion

In summary, this study has shown that increased PM_2.5_ exposure in early pregnancy and O_3_ exposure during late first trimester and over the second trimester of pregnancy were associated with incidence of gestational diabetes. Effects of O_3_ were stronger among pregnant individuals with asthma. Exposure to green space may confer protective effects in incidence of gestational diabetes through reductions in ambient air pollution. Prevention strategies aiming to reduce impacts of air pollution through increased access to green space during pregnancy should be considered. A more definitive characterization of the windows of susceptibility, especially in subgroups of the population and across mixtures of pollutants, will enhance insight into underlying mechanisms.

## Supplementary Information

**Additional file 1:** **Supplementary Table 1.** Descriptive statistics of environmental factors. **Supplementary Table 2.** Coefficient of correlation between continuous variables of interest. **Supplementary Table 3. **Adjusted cumulative hazard ratios (HRs) for individual-level covariates only and 95% confidence intervals (CIs) of gestational diabetes per interquartile range (IQR) increase in PM2.5, NO2, and O3 for the preconception period, entire pregnancy and DLM-identified sensitive windows. **Supplementary Table 4.** Adjusted mediating effects of exposures to air pollution (PM_2.5_, NO_2_ and O_3_) on the associations between the environmental exposures of interest and gestational diabetes. **Supplementary Figure 1. **Flow chart of participants exclusion. **Supplementary Figure 2. **Directed acyclic graph.

## Data Availability

The data used in this study remains confidential and cannot be shared publicly.
