# Supplementary material for: Critical windows of exposure to air pollution and gestational diabetes: assessing effect modification by maternal pre-existing conditions and environmental factors
Source: Environ Health. 2023 Mar 15;22:26. doi: 10.1186/s12940-023-00974-z (PMC10015960; doi:10.1186/s12940-023-00974-z)

**Manuscript Titre:** Critical Windows of Exposure to Air pollution and Gestational Diabetes: Assessing Effect Modification by Maternal Pre-Existing Conditions and Environmental Factors

**Authors:** Marcel Miron-Celis, Robert Talarico, Paul J. Villeneuve, Eric Crighton, David M. Stieb, Cristina Stanescu and Éric Lavigne

**Table of contents**

**Exposure ascertainment to residential exposure to green space**…………………………………………………………………….**S2**

**Supplementary Table 1.** Descriptive statistics of environmental factors. **S4**

**Supplementary Table 2.** Coefficient of correlation between continuous variables of interest **S5**

**Supplementary Table 3.** Adjusted cumulative hazard ratios (HRs) for individual-level covariates only and 95% confidence intervals (CIs) of gestational diabetes per interquartile range (IQR) increase in PM2.5, NO2, and O3 for the preconception period, entire pregnancy and DLM-identified sensitive windows……………………………………………………………………………………...**S6**

**Supplementary Table 4.** Adjusted mediating effects of exposures to air pollution (PM_2.5_, NO_2_ and O_3_) on the associations between the environmental exposures of interest and gestational diabetes **S7**

**Supplementary Figure 1.** Flow chart of participants exclusion **S8**

**Supplementary Figure 2.** Directed acyclic graph **S9**

**Exposure ascertainment to residential exposure to green space**

Two variables were used to characterize residential exposure to green space: the Normalized Difference Vegetation Index (NDVI) and the Green View Index (GVI). The NDVI is a commonly used tool to assess the level of vegetation in geographical regions [1]. It is derived through spectral reflectance measurements that are collected from Moderate Resolution Imaging Spectroradiometer (MODIS) satellite sensors. The pigment in plant leaves, chlorophyll, absorbs visible light (wavelengths 0.4–0.7 µm) for use in photosynthesis while reflecting near-infrared light (wavelengths 0.7–1.1 µm) [2]. The MODIS sensors can detect the wavelength and the intensity of light being reflected off the earth and quantify the photosynthetic capacity of the vegetation in a given area. Generally speaking, if there is much more reflected light in near-infrared wavelengths than in visible wavelengths in a given area, than the vegetation in that area is likely to be dense (e.g. forest) [2]. Conversely, if there is very little difference in the intensity of near-infrared and visible light being reflected in a given area, then the vegetation in that area is likely to be sparse or dense [2]. The NDVI is computed by dividing the difference between measured near-infrared light and visible light by the sum of these two parameters for a given area. The resulting output is always a number that ranges between -1 and +1. The value of 1 indicates the highest possible density of vegetation while the value of -1 indicates the lowest possible density of vegetation. Generally, one could interpret areas with values between 0.6 and 1 to have dense vegetation such as forests, those with values between 0.2 and 0.3 represent grasslands and shrubs while those with values of 0.1 and below tend to represent barren areas such as sand, rock, water, pavement or snow [3, 4]. NDVI data were obtained from the Canadian Urban Environmental Health Research Consortium (CANUE; <https://www.canuedata.ca/metadata.php>). Annual maximum NDVI values were computed using cloud free images Landsat 5 and Landsat 8 satellites and were linked to all six-digit postal code locations in Canada for a given calendar year.

The GVI is a more recent measure of greenness that is derived from Google Street View (GSV) panoramas. The data to compute the indicator are generally obtained by collecting images along city roads from GSV’s application program interface. The indicator can be computed using two approaches: the segmented-based GVI and the pixel-based GVI [5, 6]. The segmented-based GVI uses a standard segmentation algorithm to quantify vegetation coverage relative to non-vegetative object coverage in a given GSV photograph for specific point coordinates. Rather than using the standard segmentation algorithm, the pixel-based GVI uses the difference between green and red bands as well as green and blue bands to identify each pixel within an image as being part of vegetation. Once each pixel has been classified as being green vegetation or not, the percent of pixels representing vegetation within a given photograph for specific point coordinates is computed. Therefore, the outputs of both the segmented-based and the pixel-based GVI are the estimated proportion of vegetation cover in a given coordinate point. This study used the segmented-based GVI that was provided in CANUE because this measure tends to better predict the presence of surrounding vegetation.

References

1. Rhew IC, Vander Stoep A, Kearney A, Smith NL, Dunbar MD: Validation of the normalized difference vegetation index as a measure of neighborhood greenness. Ann Epidemiol. 2011;21 12**:**946-952; doi:10.1016/j.annepidem.2011.09.001 [doi].

2. Weier J, Herring D: Measuring Vegetation (NDVI & EVI). 2011.

3. James P, Banay RF, Hart JE, Laden F: A Review of the Health Benefits of Greenness. Curr Epidemiol Rep. 2015;2 2**:**131-142; doi:10.1007/s40471-015-0043-7 [doi].

4. Gascon M, Cirach M, Martínez D, Dadvand P, Valentín A, Plasència A, Nieuwenhuijsen MJ: Normalized difference vegetation index (NDVI) as a marker of surrounding greenness in epidemiological studies: The case of Barcelona city. Urban Forestry & Urban Greening; Special Section: Power in urban social-ecological systems: Processes and practices of governance and marginalization. 2016;19**:**88-94; doi:<https://doi.org/10.1016/j.ufug.2016.07.001>.

5. Seiferling I, Naik N, Ratti C, Proulx R: Green streets − Quantifying and mapping urban trees with street-level imagery and computer vision. Landscape Urban Plann. 2017;165**:**93-101; doi:<https://doi.org/10.1016/j.landurbplan.2017.05.010>.

6. Li X, Zhang C, Li W, Ricard R, Meng Q, Zhang W: Assessing street-level urban greenery using Google Street View and a modified green view index. Urban Forestry & Urban Greening. 2015;14 3**:**675-685; doi:<https://doi.org/10.1016/j.ufug.2015.06.006>

| Variable | Mean | Std. Dev | Median | Lower Quartile | Upper Quartile | Range | IQR |
| --- | --- | --- | --- | --- | --- | --- | --- |
| ALE | 1.12 | 3.77 | 0.35 | -0.62 | 1.52 | 45.32 | 2.14 |
| PM_2.5_, µg/m^3^ | 8.03 | 1.66 | 8.01 | 6.39 | 9.09 | 14.50 | 2.70 |
| NO_2_, ppb | 12.35 | 5.73 | 11.59 | 7.08 | 17.10 | 55.71 | 10.02 |
| O_3_, ppb | 48.49 | 4.93 | 48.39 | 44.99 | 51.99 | 69.39 | 7.00 |
| NDVI | 0.69 | 0.078 | 0.70 | 0.65 | 0.74 | 0.91 | 0.09 |
| GVI | 13.95 | 8.44 | 12.15 | 7.95 | 18.03 | 93.19 | 10.08 |
| Ambient temperature, °C | 8.30 | 2.97 | 8.32 | 6.24 | 10.57 | 31.96 | 4.33 |

**Supplementary Table 1.** Descriptive statistics of environmental factors.

Note: Std. Dev: Standard deviation; IQR: Interquartile range

|  | **GVI** | **ALE** | **NDVI** | **PM_2.5_** | **NO_2_** | **O_3_** | **Temp.** |
| --- | --- | --- | --- | --- | --- | --- | --- |
| **GVI** | 1.00 | -0.05 | 0.14 | 0.07 | -0.05 | 0.02 | 0.01 |
| **ALE** | -0.05 | 1.00 | -0.27 | 0.18 | 0.41 | -0.20 | 0.07 |
| **NDVI** | 0.14 | -0.27 | 1.00 | -0.18 | -0.26 | 0.04 | -0.01 |
| **PM_2.5_** | 0.07 | 0.18 | -0.18 | 1.00 | 0.44 | 0.25 | 0.25 |
| **NO_2_** | -0.05 | 0.41 | -0.26 | 0.44 | 1.00 | -0.27 | -0.01 |
| **O_3_** | 0.02 | -0.20 | 0.04 | 0.25 | -0.27 | 1.00 | 0.21 |
| **Temp.** | 0.01 | 0.07 | -0.01 | 0.25 | -0.01 | 0.21 | 1.00 |

**Supplementary Table 2.** Coefficient of correlation between continuous variables of interest.

**Supplementary Table 3.** Adjusted^*^ cumulative hazard ratios (HRs) and 95% confidence intervals (CIs) of gestational diabetes per interquartile range (IQR) increase in PM_2.5_, NO_2_, and O_3_ for the preconception period, entire pregnancy and DLM-identified sensitive windows.

| Pollutant | HR (95% CI) |
| --- | --- |
| PM_2.5_ (per IQR = 2.7 µg/m^3^ increase) |  |
| Preconception period | 1.02 (0.99 – 1.04) |
| Pregnancy period | 1.06 (0.98 – 1.11) |
| Sensitive windows | 1.09 (1.03 – 1.12) |
|  |  |
| NO_2_ (per IQR = 10.0 ppb increase) |  |
| Preconception period | 1.05 (0.91 – 1.21) |
| Pregnancy period | 0.99 (0.85 – 1.16) |
| Sensitive windows |  |
|  |  |
| O_3_ (per IQR = 7.0 ppb increase) |  |
| Preconception period | 1.06 (1.02 – 1.10) |
| Pregnancy period | 1.05 (1.00 – 1.09) |
| Sensitive windows | 1.10 (1.05 – 1.14) |

^*^Adjusted for individual-level covariates only: maternal age, parity, maternal smoking status, prepregnancy body mass index, month of birth and year of birth.

**Supplementary Table 4.** Adjusted^*^ mediating effects of exposures to air pollution (PM_2.5_, NO_2_ and O_3_) on the associations between the environmental exposures of interest and gestational diabetes.

| Exposures | HR (95% CI) |
| --- | --- |
| GVI |  |
| Natural direct effect | 0.95 (0.93–0.98) |
| Natural indirect effect | 0.99 (0.98–0.99) |
| Total effect | 0.94 (0.92–0.96) |
| % of effect explained by mediator (95% CI) | 20.1 (17.6–22.6) |
|  |  |
| NDVI |  |
| Natural direct effect | 0.92 (0.89–0.95) |
| Natural indirect effect | 1.00 (0.99–1.01) |
| Total effect | 0.92 (0.89–0.95) |
| % of effect explained by mediator (95% CI) | 1.4 (1.9–4.7) |
|  |  |
| ALE |  |
| Natural direct effect | 0.98 (0.97–0.99) |
| Natural indirect effect | 1.00 (1.00–1.00) |
| Total effect | 0.98 (0.97–0.99) |
| % of effect explained by mediator (95% CI) | 4.6 (3.6–5.6) |

^*^Adjusted for maternal age, parity, maternal smoking status, prepregnancy body mass index, weekly ambient temperatures, month of birth, year of birth, residing in the Greater Toronto Area, community size, deprivation quintiles, instability quintiles, dependency quintiles and ethnic quintiles.

**Supplementary Figure 1.** Flowchart of the inclusion and exclusion of the study population.


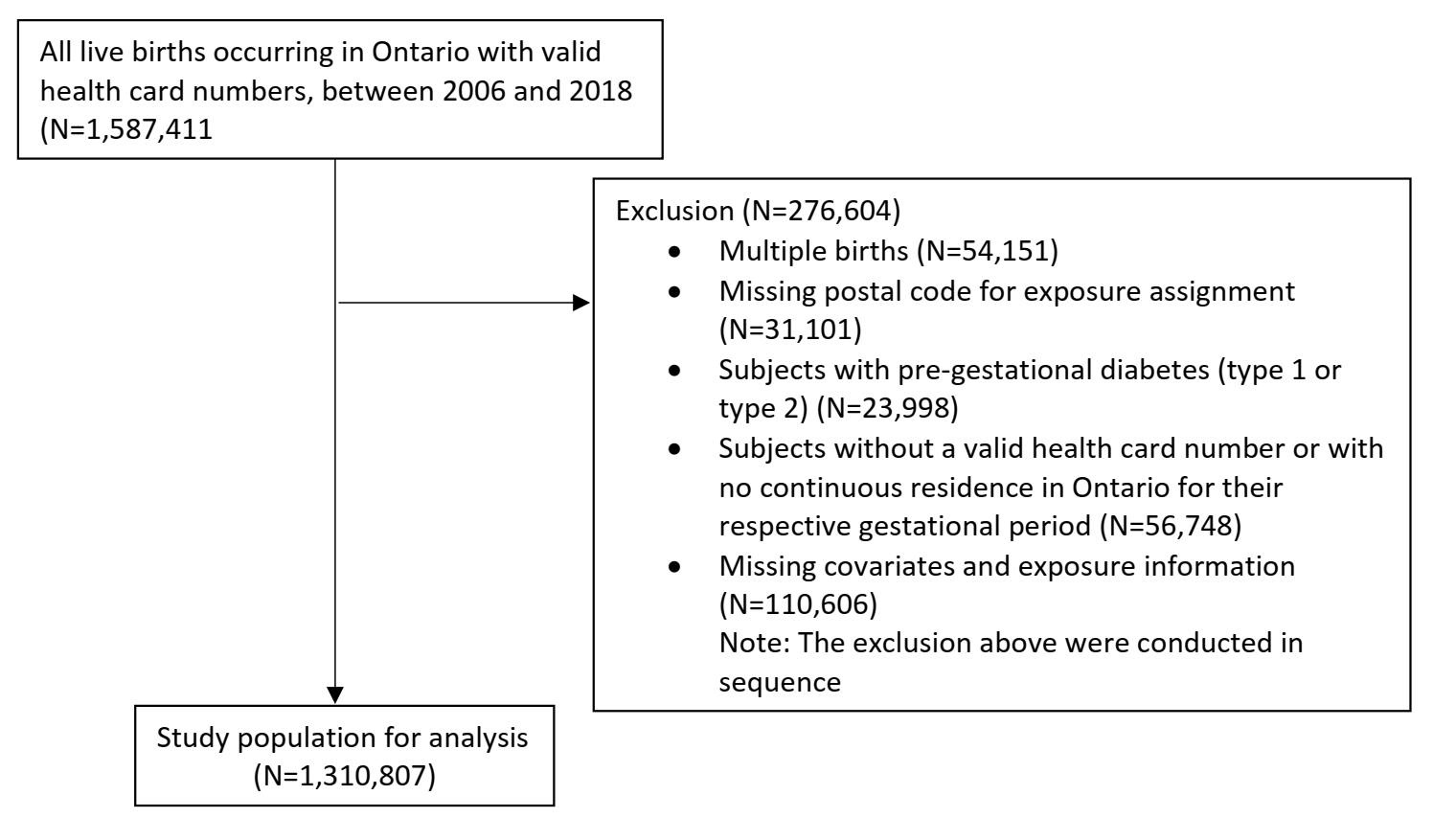


**Supplementary Figure 2.** Directed acyclic graph for estimating the direct effect of residential of air pollution on gestational diabetes. Parameters in red are potential confounding factors and parameters in grey are unmeasured variables. Green line: causal path. According to the DAG, the minimal sufficient adjustment for estimating the total effect of air pollution exposure on gestational diabetes is: maternal age, parity, maternal smoking status, prepregnancy body mass index, weekly ambient temperatures, month of birth, year of birth, residing in the Greater Toronto Area, community size, deprivation quintiles, instability quintiles, dependency quintiles and ethnic quintiles. (Color should be used for Supplementary Figure 1)


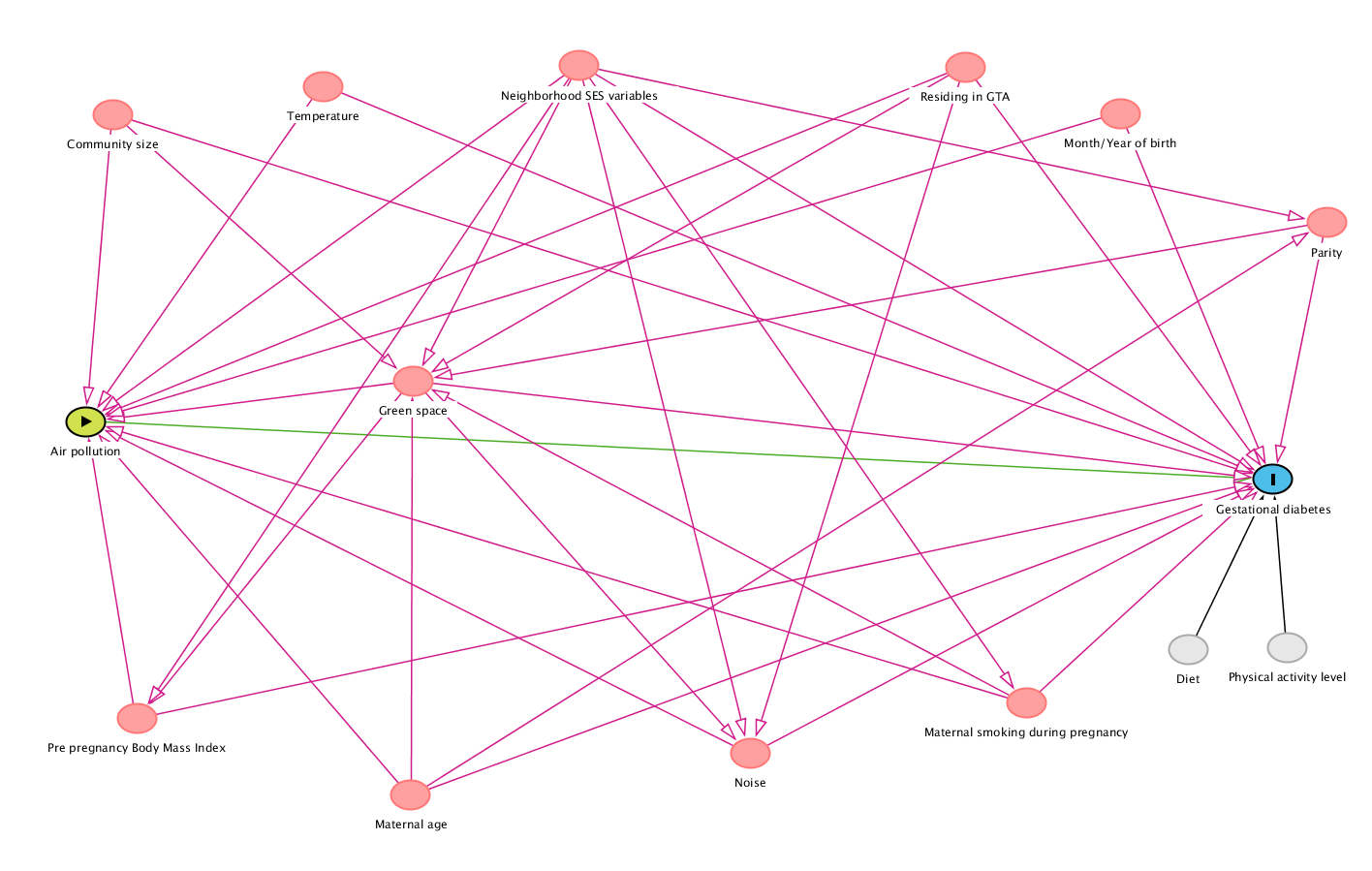

Supplement: Supplementary file 1 — Additional file 1: Supplementary Table 1. Descriptive statistics of environmental factors. Supplementary Table 2. Coefficient of correlation between continuous variables of interest. Supplementary Table 3. Adjusted cumulative hazard ratios (HRs) for individual-level covariates only and 95% confidence intervals (CIs) of gestational diabetes per interquartile range (IQR) increase in PM2.5, NO2, and O3 for the preconception period, entire pregnancy and DLM-identified sensitive windows. Supplementary Table 4. Adjusted mediating effects of exposures to air pollution (PM2.5, NO2 and O3) on the associations between the environmental exposures of interest and gestational diabetes. Supplementary Figure 1. Flow chart of participants exclusion. Supplementary Figure 2. Directed acyclic graph. [file 12940_2023_974_MOESM1_ESM.docx]
